# Migratory vasodilatation of cerebral arteries in MELAS episodes: a case report and literature review

**DOI:** 10.3389/fimmu.2026.1706012

**Published:** 2026-02-05

**Authors:** Ying Luo, Quanhong Chu, Taochun Yu, Xiaoqing Lu, Yanmei Wang, Jie Li, Lingfeng Wu, Yaoyao Shen

**Affiliations:** 1 Department of Neurology, Jiangxi Chest Hospital, The Third Affiliated Hospital of Nanchang Medical College, Nanchang, Jiangxi, China; 2 Department of Neurology, Jiangxi Provincial People’s Hospital, The First Affiliated Hospital of Nanchang Medical College, Nanchang, Jiangxi, China; 3 Department of Neurology, Xiangya Hospital, Central South University, Jiangxi, National Regional Center for Neurological Diseases, Nanchang, Jiangxi, China; 4 Department of Rehabilitation Medicine, Affiliated Hospital of Jiujiang University, Jiujiang, Jiangxi, China

**Keywords:** CASPR2, cerebral blood flow, MELAS, stroke-like episodes, vasodilatation

## Abstract

Mitochondrial encephalomyopathy with lactic acidosis and stroke-like episodes (MELAS) is the commonest inherited mitochondrial disorder. Dilation of the major cerebral arteries is seldom mentioned in MELAS because magnetic resonance angiography (MRA) usually shows normal findings. Here, we described a 24-year-old male patient with MELAS who had migratory vasodilatation of cerebral arteries on MRA and positive antibodies against contactin-associated protein-like-2 in serum during stroke-like episodes (SLEs). Moreover, a total of 18 MELAS cases were included in this literature review. MELAS with cerebrovascular dilatation was mainly seen in young adults and the commonest clinical manifestations were visual symptoms, headache, and seizures. Vasodilatation predominantly occurred in the acute phase of SLEs. Dilation of the major cerebral artery mainly happened in the posterior cerebral artery and the middle cerebral artery. The region of increased cerebral blood flow (CBF) always corresponded to the range of vascular supply of the dilated cerebral artery. Multimodal neuroimaging evaluation is very important for the diagnosis of mitochondrial diseases. Migratory vasodilation of cerebral arteries may be a specific imaging sign of MELAS. Dilation of cerebral artery together with increased CBF not only indicates the clinical onset, but also implies an upcoming stroke-like attack.

## 1 Introduction

Mitochondrial encephalomyopathy with lactic acidosis and stroke-like episodes (MELAS) is a progressive, multisystem affected inherited mitochondrial disease caused by genetic mutations in mitochondrial DNA and/or nuclear DNA, with high clinical heterogeneity (1, 2). The onset of MELAS is often before age of 15 years, and the main neurological manifestations include stroke-like episodes (SLEs), seizures, dementia, recurrent headache, deafness, and cortical blindness (3, 4). If muscle biopsy and genetic detection are not performed, MELAS may easily be misdiagnosed as cerebral infarction, viral encephalitis, or autoimmune encephalitis (AE). Approximately 80% of MELAS patients are caused by the m.3243A>G mutation that affects the electron transport chain (ETC) complex subunits (especially the complex I and complex IV) by reducing mitochondrial protein synthesis, thereby resulting in deficiency of the mitochondrial energy production (2, 5). Insufficient energy supply not only leads to multi-organ dysfunction, but also gives rise to mitochondrial proliferation in smooth muscle and endothelial cells of small blood vessels causing impairment of blood perfusion in microvasculature (2). Although macroangiopathy is rarely observed in patients with MELAS, there is still a debate about the changes of the large cerebral arteries in this entity. Segmental stenosis of the major cerebral arteries on magnetic resonance angiography (MRA) has been described in MELAS episodes, and some of the narrowing vessels may be reversible during follow-up (6–9). On the contrary, dilatation of cerebral arteries can also be found in the acute phase of SLEs (10). Macroangiopathy, therefore, may also play an essential role in hemodynamic regulation during MELAS episodes. Here, we report an interesting MELAS case caused by m.3243A>G mutation in the *MT-TL1* gene. It’s worth noting that migratory vasodilatation of cerebral arteries can be visualized on MRA in a series of SLEs. Moreover, we detect positive antibodies against contactin-associated protein-like-2 (CASPR2) in serum during the acute and remission stages of the first episode. To the best of our knowledge, this is the second reported case of MELAS with positive anti-CASPR2 antibody. In addition, we perform a review for similar cases documented in the literature in order to deepen our awareness of the dilation of cerebral artery in MELAS.

## 2 Case description

**Figure 1:**
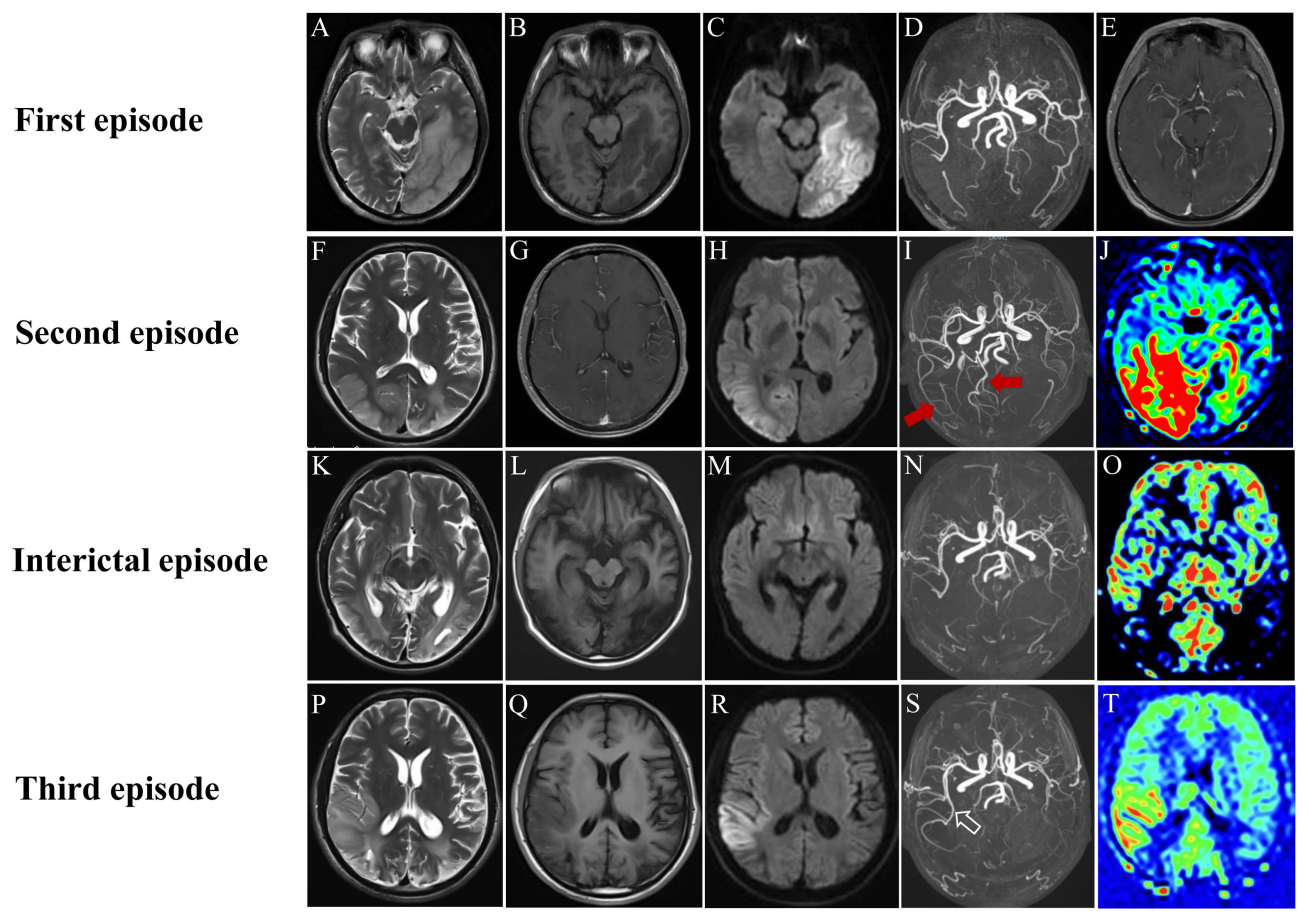
Neuroimaging findings of our case: In the first episode, MRI revealed a stroke-like lesion (SLL) in the left temporo-occipital lobe **(A-C)**, with focal leptomeningeal enhancement **(E)**. MRA showed dilatation of the left distal PCA **(D)** (white arrow); In the second episode, MRI demonstrated a new SLL in the right temporo-occipital lobe, without enhancement **(F-H)**. MRA and ASL disclosed dilatation of right cerebral arteries (red arrows) and hyperperfusion in the right temporo-occipital lobe, respectively **(I, J)**; Follow-up neuroimaging after the second episode illustrated cortical atrophy, resolution of dilated arteries, and hypoperfusion in affected regions **(K-O)**; In the third episode, MRI showed a new lesion in the right temporo-insular lobe **(P-R)**. The distal right MCA was dilated again (blank arrow) with hyperperfusion in its vascular supply regions **(S, T)**.

**Figure 2:**
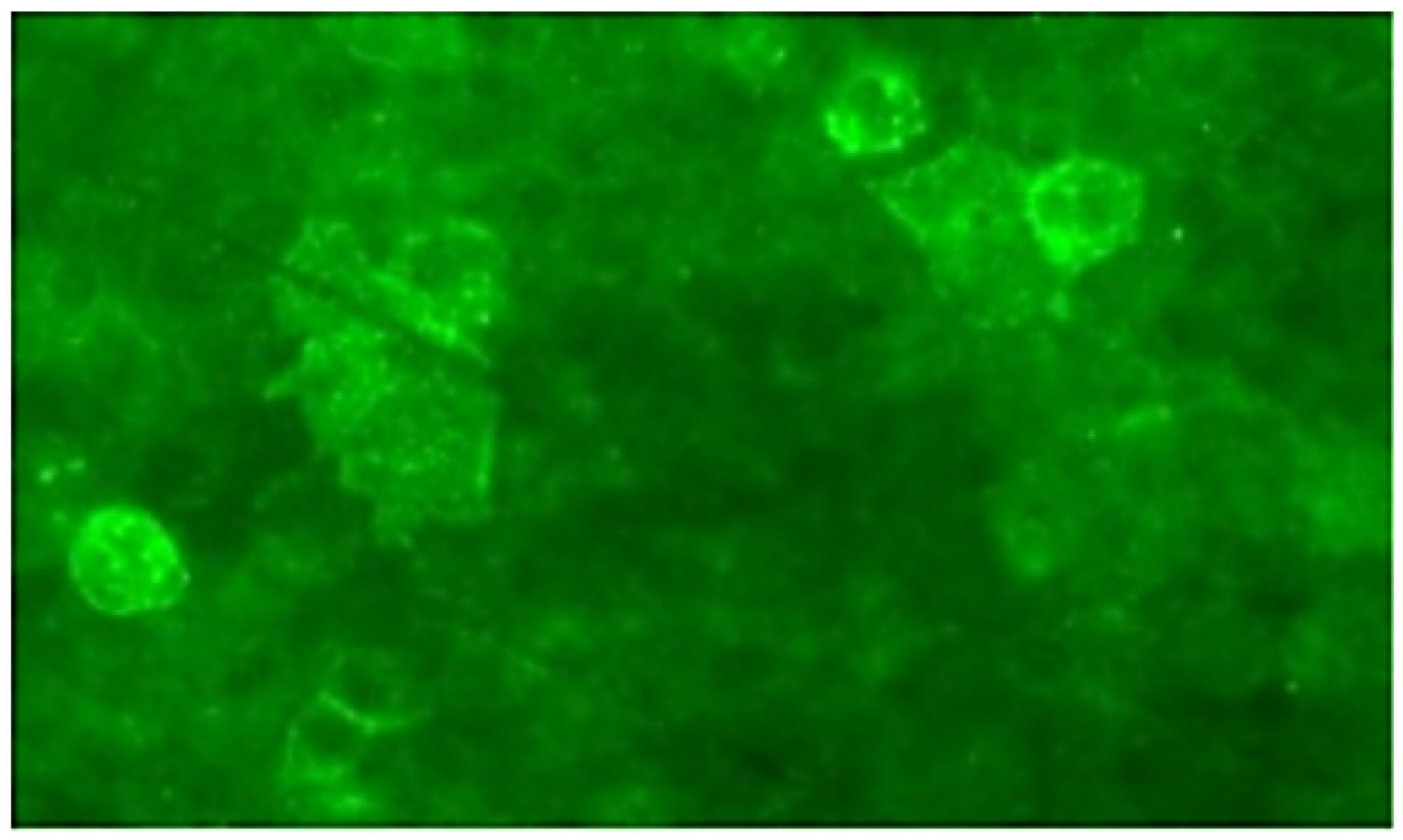
Immunofluorescence of anti-Caspr2 antibodies in the patient’s serum.

On April 19, 2021, a previously healthy 24-year-old man was admitted to our department due to a 7-day history of headache, blurred vision, and cognitive decline. Initially, he complained of persistent headache, mainly in the left temporo-occipital area, accompanied by blurred vision and low-grade fever (ranging from 37.3 to 38.0°C). Two days later, his body temperature dropped to normal range. However, the patient noticed a decline in his memory, and had slow response when he was communicating with others. His past medical history was unremarkable except for appendectomy and chronic hepatitis B. He was thin since childhood and his academic performance was relatively poor during school time. The exercise tolerance of this patient is not as good as that of his peers. On admission, his vital signs were stable with a body mass index of 18.0 Kg/m^2^ (height 160 cm, weight 46 Kg). Neurological examination revealed disorientation in time and place, poor computing power, memory impairment, and visual field defect on his right side. Testing for muscle strength, muscle tone, and tendon reflexes of four limbs showed normal findings. Mini-Mental State Examination (MMSE) score was 17. Laboratory examinations, including complete blood count, biochemistry, C-reactive protein, thyroid hormones, tumor markers, coagulation function, and antinuclear antibody spectrum, were all within normal limits. Antibodies against human immunodeficiency virus and syphilis were negative in serum. On the same day, brain magnetic resonance imaging (MRI) demonstrated abnormal signal intensities in the left temporo-occipital lobe with distinct cortical and subcortical swelling (**Figures 1A–C**). Slight vasodilatation of the left distal posterior cerebral artery (PCA) was visualized on MRA (**Figure 1D**). T1-weighted post-contrast image disclosed multifocal leptomeningeal enhancement along the sulci of left temporo-occipital lobe (**Figure 1E**). On the next day, lumbar puncture was performed subsequently, showing increased cell count (20 cells/mm3), with normal opening pressure, protein, glucose, and chloride. Microbiological investigations, consisting of bacterial culture, acid-fast bacilli smear, T-spot, India Ink preparation, were all negative. AE-related antibody testing yielded anti-CASPR2 antibody was positive in serum with a titer of 1:32 (**Figure 2**), but negative in cerebrospinal fluid (CSF). Hence, he was initially diagnosed with anti-CASPR2 encephalitis, and administrated with oral prednisolone (60 mg/d followed by gradual taper for a total course of 6 months) and intravenous immunoglobulin (0.4 g/Kg/d for 5 days). On day 10 after admission, his cognitive impairment and blurred vision improved partially. He was discharged on May 7, 2021. One month later, the patient visited to our outpatient department for retesting AE-related antibody, and the result showed the titer of anti-CASPR2 antibody in serum dropped to 1:10.

**Figure 3:**
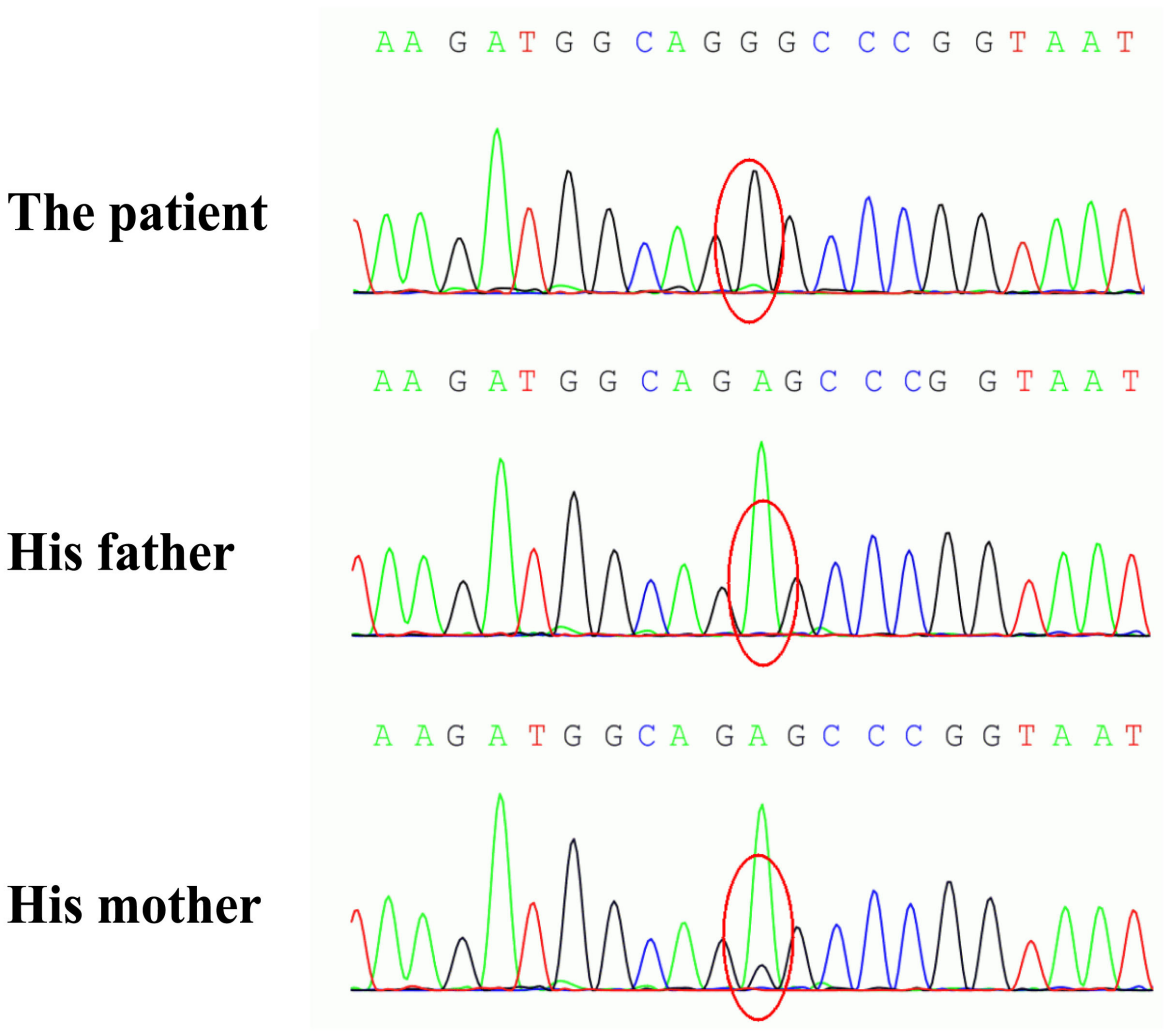
The genetic analysis of the patient and his parents. A pathogenic m.3243A>G mutation in the *MT-TL1* gene was identified in the patient and his mother.

On December 3, 2021, the patient came to our hospital again because of 6-hour history of paroxysmal occipital headache and loss of vision in both eyes, accompanied by nausea, vomiting, and fever (a maximum temperature of 38.9°C). He was unable to recognize people and objects, but could slightly perceive their outlines in the daytime. Neurological examination demonstrated bilateral blindness (only light perception), spontaneous nystagmus, memory impairment, neck stiffness, and positive Kernig’s sign. Pupils were 3 mm in each eye and equally reactive to light, without relative afferent pupillary defect. Fundus examination showed normal findings and there was no other focal neurological deficit. Serological investigations were unremarkable except for elevated blood lactate (6.43 mmol/L) in blood gas analysis. On the day of the second admission, brain MRI revealed a new lesion located in the right temporo-occipital lobe, with hyperintense on T2-weighted image (T2WI) and diffusion-weighted image (DWI) (**Figures 1F, H**). There was no enhancement within the abnormal area on T1-weighted post-contrast image (**Figure 1G**). Meanwhile, MRA illustrated apparent vasodilatation of right cerebral arteries, including the PCA and the middle cerebral artery (MCA) (**Figure 1I**). Hyperperfusion was noted in the right temporo-occipital lobe on arterial spin labeling (ASL) image (**Figure 1J**). Electroencephalography (EEG) exhibited increased slow-wave activity. The CSF analyses yielded elevated lactate (4.57 mmol/L), with normal cell count and protein. Electromyography and muscle biopsy were performed for the patient, but the results was normal. Urine samples were collected from the patient and his parents, and the genetic testing results demonstrated the patient and his mother had a pathogenic mutation in the *MT-TL1* gene (m.3243A>G) (**Figure 3**). Therefore, a modified diagnosis of MELAS was established, and empirical therapies, encompassing L-arginine, coenzyme Q10, B vitamins, and levocarnitine, were started. On December 28, 2021, he was discharged without any improvement of visual acuity. Follow-up MRI, on October 3, 2022, disclosed cortical atrophy in bilateral temporo-occipital lobes without restricted diffusion (**Figures 1K–M**). MRA and ASL showed the resolution of dilated right cerebral arteries and significant hypoperfusion in bilateral temporo-occipital lobes, respectively (**Figures 1N, O**).

On January 27, 2024, he experienced a third episode. Sometimes, his left limbs were out of control accompanied by insensitive cold and heat sensations. These symptoms were paroxysmal, transient and stereotypical, suggesting a simple partial seizure. He was blind in both eyes and could only perceive light, with obvious learning and memory deficits. He always manifested as obvious anxiety and depression when confronted with frustrations. Brian MRI revealed a new stroke-like lesion in the right temporal and insular lobes as well as encephalomalacia in bilateral temporo-occipital lobes (**Figures 1P–R**). The distal right MCA was dilated again (**Figure 1S**), and hyperperfusion was seen in its corresponding vascular territories (**Figure 1T**). After treated with oxcarbazepine, focal seizures subsided gradually. He was discharge 7 days later.

## 3 Discussion

We reported a 24-year-old male patient with MELAS caused by m.3243A>G mutation in the *MT-TL1* gene. Migratory vasodilatation of cerebral arteries during SLEs could be observed by dynamic MRA examinations. To further summarize the clinical and imaging features of MELAS patients with dilatation of cerebral arteries, we performed a comprehensive literature review. Web of Science, PubMed, and Embase databases were systematic searched from inception to January 2025. The title/abstract and all fields combination search phrases were “MELAS or mitochondrial encephalomyopathy with lactic acidosis and stroke-like episodes,” and “artery dilation or vasodilatation”. Reference lists of the selected articles were reviewed for potential additional cases. A total of eighteen MELAS patients with cerebral artery dilation (including our case), consisting of fourteen males (14/18, 77.8%) and four females (4/18, 22.2%), were incorporated in this literature review. The clinical and neuroimaging features of selected cases was summarized in **Table 1**. The age of onset ranged from 13 to 57 years, and the mean age was 26.1 years. Genetic testing was performed for all included patients, and all gene mutations were caused by m.3243 A > G except for 1 resulting from m.13,513 G > A. Vasodilatation not only occurred in the acute phase (16/18, 88.9%) of SLEs, but also happened in the preclinical phase (2/18, 11.1%). Dilation of cerebral artery was predominately occurred in the MCA (14/18, 77.8%) and the PCA (10/18, 55.6%), but rarely in the anterior cerebral artery (ACA) (2/18, 11.1%) and the internal carotid artery (ICA) (1/18, 5.6%). Multiple vessels dilation and single vessel dilation were found in 10 and 8 cases, respectively. In this review, 16 MELAS patients presented with a wide spectrum of neurological manifestations in the acute phase of SLEs, and the commonest ones were visual symptoms (12/16, 75%), headache (11/16, 68.8%) and seizures (7/16, 43.8%). Other neurological symptoms included cognitive decline, limbs weakness, blurred speech, hearing loss, mental and behavior disturbance, disturbance of consciousness, and ataxia. Only 2 patients experienced fever during MELAS episodes. Almost all stroke-like lesions were located in unilateral cerebral hemisphere. Involved cerebral cortex include parietal (10/16, 62.5%), temporal (10/16, 62.5%), and occipital (10/16, 62.5%) lobes. It should be emphasized that the frontal lobe was preserved in all cases. The number of cases of multiple lobes involvement (9/16, 56.2%) was slightly more than that of single lobe involvement (7/16, 43.8%). In the acute and preclinical phases of SLEs, fifteen patients had increased cerebral blood flow (CBF). The region of increased CBF always corresponded to the range of vascular supply of the dilated cerebral artery. The remaining 3 patients had no relevant records.

**Table 1:** The clinical data of MELAS patients with cerebral artery vasodilation.

Case No.	Age (yrs), Sex	Gene mutation	Stage of disease	Clinical presentation	Dilated artery	Stroke-like Lesion	HP
1 (9)	32, M	m.3243 A > G	Acute phase	Headache, blurred vision	Right MCA and PCA	Right temporo-occipital lobe	NA
2 (9)	57, F	m.3243 A > G	Acute phase	Mental and behavior disturbance, seizures	Right MCA	Right temporo-parietal lobe	NA
3 (10)	22, F	m.3243 A > G	Acute phase	Headache, blurred vision, hemianopia, seizures	Left MCA and PCA	Left occipital lobe	+
4 (11)	41, M	m.3243 A > G	Acute phase	Headache, seizures, hemianopia	Right MCA and PCA	Right parieto-occipital lobe	+
5 (11)	18, M	m.3243 A > G	Acute phase	Headache, hemianopia	Left PCA	Left occipital lobe	+
6 (11)	24, M	m.3243 A > G	Acute phase	Left arm weakness	Right MCA	Right temporal lobe	+
7 (11)	20, M	m.3243 A > G	Acute phase	Headache, hearing loss, aphasia, weakness	Left MCA	Left temporo-parietal lobe	+
8 (11)	22, M	m.3243 A > G	Acute phase	Headache, vomiting, blurred vision, seizures, cognitive decline	Bilateral MCAs	Right temporal lobe	+
9 (11)	13, M	m.13,513 G > A	Acute phase	Blurred vision, seizures, ataxia	Left MCA, Right ACA	Left parietal lobe, Right parietal lobe	+
10 (11)	22, M	m.3243 A > G	Acute phase	Headache, blurred vision, fever	Left MCA, ACA and PCA	Left parieto-occipital lobe	+
11 (11)	20, M	m.3243 A > G	Acute phase	Headache, hemianopia	Right PCA	Right occipital lobe	+
12 (11)	27, M	m.3243 A > G	Acute phase	Headache, blurred vision, blurred speech, disturbance of consciousness	Left MCA	Left temporo-parietal lobe	+
13 (11)	14, F	m.3243 A > G	Acute phase	Hemianopia, seizures, weakness	Left MCA and PCA, Right MCA	Left parietal, temporal, and occipital lobes	+
14 (11)	18, M	m.3243 A > G	Acute phase	Slurred speech, hearing loss, cognitive decline	Left MCA and PCA, Right MCA	Left parietal, temporal, and occipital lobes	+
15 (11)	20, F	m.3243 A > G	Preclinical phase	–	Right MCA and PCA	–	+
16 (12)	22, M	m.3243 A > G	Preclinical phase	–	Left MCA	–	+
17 (13)	53, M	m.3243 A > G	Acute phase	Cortical blindness, headache, seizures, hearing loss, quadriplegia	Bilateral ICAs	Bilateral parietal, temporal, and occipital lobes	NA
18 (our case)	24, M	m.3243 A > G	Acute phase	Headache, blurred vision, cognitive decline, fever	Left PCA	Left temporo-occipital lobe	+

Abbreviations: No. Number, F female, M male, yrs years, MCA middle cerebral artery, ACA anterior cerebral artery, PCA posterior cerebral artery, ICA internal carotid artery, HP hyperperfusion, NA not available.

MELAS, one of the most frequent inherited mitochondrial disorders, was first described by Pavlakis in 1984 (2). Although the majority of MELAS patients show maternal inheritance, there are still many sporadic cases. Even in the same family with maternal inheritance, the clinical manifestations of MELAS vary widely among individuals. In many MELAS families, the probands present with typical symptoms, but their mothers are often oligosymptomatic (fatigability, deafness, diabetes mellitus, and short stature) or asymptomatic (14, 15). As our case reported here, the patient suffered from recurrent SLEs, whereas his mother was asymptomatic in spite of having the same mutation in the *MT-TL1* gene (m.3243A>G). The prevalence of MELAS was estimated to be 0.2 per 100,000 in Japan, but relatively high in northern Finland, approximately 16~18 per 100,000 (2, 16, 17). While both men and women are equally susceptible to this disease, only women can transmit MELAS to the next generation because mitochondria that are carried in the tails of sperm cells are shed outside the zygote during fertilization. The age of onset spans a wide range from 2 to 80 years and about 75% of MELAS patients have undergone an initial attack before the age of 20 years (18). MELAS is a multi-organ affected disease with varied clinical manifestations including SLEs, headache, seizures, cognitive impairment, hearing loss, lactic acidemia, myopathy, diabetes, and short stature. In this review, MELAS mainly occurs in young adults, with a mean age of 26.1 years. Late-onset MELAS (age of onset>40 years) is relatively rare, accounting for 16.7% of our selected cases. The frequent neurological symptoms are visual symptoms (75%), headache (68.8%), and seizures (43.8%). Visual symptoms encompass blurred vision, hemianopsia, and cortical blindness. The reason for high occurrence rate of visual symptoms is that the occipital lobe is more susceptible to damage than other cerebral lobes during MELAS episodes. Previous studies have shown that compared with general population there is a higher incidence of migraine in patients with mitochondrial disorders (19, 20). Besides, MELAS patients with migraine are more likely to encounter epilepsy than that without migraine (19). Epilepsy is one of the core clinical features of MELAS, consisting of partial seizures (simple/complex partial seizure or secondarily generalized) and generalized seizures (myoclonic or tonic-clonic) (21). The most common type is partial seizures indicating a focal origin (22). Nonconvulsive status epilepticus has also been documented in MELAS patients (23).

MRI provides an important clue for diagnostic evaluation of mitochondrial disorders and helps clinicians to distinguish MELAS form other diseases, such as cerebral infarction. Acute ischemic stroke is characterized by sudden deprivation of CBF to a brain region. The infarction lesion often demonstrates distinct hypoperfusion, and the corresponding supplying artery may have a focal stenosis or occlusion on MRA. Previous clinical researches trend to focus on the neuroimaging characteristics of SLLs, which are observed in the majority of MELAS patients and closely related to focal neurological symptoms, particularly SLEs. Typical MRI feature of MELAS is cortical lesions distributed along gyri mimicking multifocal infarction, characterized by acute diffusion restriction, subacute cortical laminar necrosis, and eventual chronic cortical atrophy. SLLs have a preference for the posterior regions of the brain, such as occipital, parietal or temporal lobe, but rarely emerge in basal ganglia, thalamus, cerebellum, brainstem or frontal lobe (24, 25). Lesions are generally limited to the cortex and subcortical white matter, yet sometimes can extend to the deep white matter. Since the distribution of SLLs does not follow vascular territories, it’s not difficult to distinguish MELAS from cerebral infarction. In acute phase, brain MRI shows hyperintensity in cerebral cortex on T2WI/FLAIR and DWI, accompanied by cortex swelling and sulcal effacement. In addition, parts of cortical lesions reveal a patchy or linear enhancement on T1-weighted postcontrast images owing to the breakdown of blood-brain barrier and increased CBF in focal regions. In subacute phase, SLLs may develop into cortical laminar necrosis, displaying gyriform hyperintensity on T1-weighted image (T1WI) and hypointensity on T2WI/T2FLAIR (26). In chronic phase, the volume of the affected brain tissue gradually shrinks over time and those cortical lesions finally evolve into encephalomalacia (27). ASL, a non-invasive neuroradiological technique, is utilized to evaluating cerebral perfusion. In acute phase of SLEs, distinctly increased CBF is noted within the range of SLLs on ASL images. With acute SLLs gradually develop into chronical encephalomalacia, the CBF in affected areas persistently decreases, eventually becoming hypoperfusion. Actually, focal hyperperfusion has already appeared several months before the clinical onset of SLEs (also known as preclinical phase) (12, 28). We believe such dynamic evolution of perfusion on ASL can essentially distinguish MELAS from cerebral infarction. Furthermore, hyperperfusion in preclinical phase may be a potential predictor for the upcoming clinical onset of SLEs. Another non-invasive imaging technique, proton magnetic resonance spectroscopy (^1^H-MRS), is widely used as a tool for assessing the metabolic and biochemical changes of brain. The typical ^1^H-MRS feature of MELAS is a high, inverted lactate peak in the regions of interest, reflecting an anaerobic metabolism. There is a positive correlation between lactate levels in ^1^H-MRS and that in CSF in MELAS patients (29). Elevated lactate in affected region tends to gradually resolve as the lesion progressively evolve into cerebral atrophy. Several studies have reported that high levels of lactate peaks on ^1^H-MRS are related to increased disease severity (30, 31).

Previously, two mainstream theories—mitochondrial angiopathy and mitochondrial cytopathy—have been proposed to explain the pathophysiologic mechanism of SLEs. The former is mitochondrial dysfunction in smooth muscle and endothelial cells of the cerebral blood vessels, while the latter is intracellular metabolic disorders owing to mitochondrial dysfunction (32). Regarding the changes of vascular structure in MELAS previous studies have found that there are large numbers of abnormal mitochondria in the smooth muscle and endothelial cells of cerebral arterioles, which are the main resistance vessels of regulation of CBF, especially pial arterioles with a diameter of less than 250 μm. Moreover, polymerase chain reaction restriction fragment length polymorphism analysis has confirmed that the proportion of mutated mtDNA in the walls of the pial and cortical arterioles is the highest. What’s more, widespread respiratory chain deficiency exists in the cerebral blood vessels (33). Based on above-mentioned findings, it can be inferred that primary mitochondrial dysfunction-induced small cerebral vascular impairment plays an important role in the pathogenesis of SLEs. An alternative theory is that mitochondrial dysfunction leads to intracellular metabolic disorder, including increased anaerobic metabolism, deficiency of energy production, hyperproduction of oxygen free radicals, and accumulation of lactic acid, further resulting in enhanced excitability of neurons, increased capillary permeability, and dilation of the capillaries (32). A combination of mitochondrial angiopathy and mitochondrial cytopathy contributes to cortical necrosis, vasogenic edema, hemodynamic disorder, ultimately triggering a stroke-like attack. However, macroangiopathy is seldom mentioned in MELAS as MRA usually reveals normal findings. A systematic review has showed that dilation of major cerebral arteries was seen in 37% of MELAS patients, and the commonest involved vessel was the MCA (6). Vasodilatation occurred in 88% of them in acute phase of SLEs, and such vascular change could be reversible during follow-up. Similar results can be obtained from our literature review. Vasodilatation mainly occurs in the acute phase of SLEs, but may also emerge several months before the clinical onset of SLEs. Dilation of cerebral artery can be observed in both anterior and posterior circulations, yet the PCA and the MCA are the most common. Dilation of multiple vessels is more frequent than that of single vessel. We recommend a dynamic angiographic monitoring at different periods of SLEs in order to better understand its pathophysiological processes. Preclinical vasodilation with hyperperfusion (generally several months before clinical onset) is highly suggestive of an upcoming stroke-like attack. The preferential distribution of lesions in the occipital lobe is attributed to the high metabolic demand in this region.

At present MELAS is still incurable and the treatment strategy primarily focuses on multidisciplinary management around symptomatic and supportive care. It is known that several supplementations, including vitamins, cofactors, and antioxidants, may be helpful according to limited clinical trials (2). Riboflavin, also called vitaminB2, plays a role in supporting complex I activity in the ETC. A daily dose of 50–400 mg of riboflavin intake may be salutary, especially in patients with complex I deficiencies. In addition, coenzyme Q10 as electron carrier in the mitochondrial ETC is crucial for maintaining oxidative phosphorylation and energy production. Treatment with coenzyme Q10 has beneficial effects on muscle weakness, fatigability, and lactate level in patients with MELAS. Moreover, L-carnitine can also promote energy production by transporting long-chain fatty acids into the mitochondria for β-oxidation. It enhances mitochondrial function by improving fatty acid metabolism and reducing the accumulation of toxic acyl compounds. The use of L-arginine is mainly based on the hypothesis of mitochondrial angiopathy. During SLEs, the decreased level of L-arginine affects the function of endothelial cells, thereby resulting in dysfunction of the dilation of cerebral arterioles and focal cerebral ischemia. Supplementing L-arginine to MELAS patients can increase the production of nitric oxide, which can improve endothelial cell function and vascular regulatory function, potentially reducing the frequency and severity of SLEs (34). Gene-editing therapy is expected to correct the underlying genetic defects, but is still on the way. It is also hoped that more treatments will be developed to improve the life quality of MELAS patients. In our case, a special neuroimaging sign, migratory vasodilation of cerebral arteries, has attracted our attention. The patient underwent a total of three episodes. Whenever a new episode occurs, previous dilated arteries disappeared and are replaced by vasodilatation of other cerebral arteries. Migratory vasodilation of cerebral arteries may be a neuroimaging hallmark of MELAS. The reason for this phenomenon may be that elevated vasorelaxation factors (such as nitric oxide) cause relaxation of the smooth muscle cells and vasodilation of the cerebral vessels (35). We speculate that vasodilatation may also be a compensatory state due to insufficient energy supply during SLEs. In addition, we also observe a relatively symmetric cortical involvement in the third episode, reflecting anatomical symmetry of high metabolic demands in the brain. Last but not least, we should not ignore to discuss the relationship between MELAS and anti-CASPR2 antibody. To our knowledge, CASPR2 is a cell adhesion protein that is widely expressed on axons of the central and peripheral nervous system, such as the cortex, basal ganglia, limbic system, thalamus, and sensory organs (36). Anti-CASPR2 antibody is commonly associated with limbic encephalitis, Morvan syndrome, neuromyotonia, and painful neuropathy (37). In patients with positive anti-CASPR2 antibody, 53.1% of them have abnormal brain MRI findings, which mainly include T2WI/FLARI hyperintensity in the medial temporal lobe, hippocampal atrophy, and mesial temporal sclerosis/hippocampal sclerosis (37). Though some cases of anti-CASPR2 antibody-associated AE have MRI abnormalities in the basal ganglia, cerebral cortex and subcortical white matter, lesion outside the temporal lobe is extremely rare (37–40). Previously, there has been only one report of MELAS patient with anti-CASPR2 antibody (41). That patient has abnormalities in bilateral lateral temporal lobes on MRI and positive antibodies against CASPR2 in serum (1:32). His poor response to glucocorticoid together with lamellar necrosis of the lateral temporal lobe doesn’t seem to meet the diagnosis of anti-CASPR2 antibody-associated AE. There are several differences between our patient and the foregoing one. In our case, the stroke-like lesion has extended to the medial temporal lobe during the first attack. To verify the reliability of anti-CASPR2 antibody, we have retested AE-related antibodies. We think that the emergence of anti-CASPR2 antibody in MELAS results from antigenic exposure following the impairment of the medial temporal lobe rather than direct pathogenic antibody. Whether immunologic mechanisms truly participate in the pathogenesis of MELAS remains unclear. Further clinical researched are needed to explore the significance of anti-CASPR2 antibody in MELAS patients.

## 4 Conclusion

In summary, the clinical manifestations of MELAS are complex and varied. Without muscle biopsy and genetic testing, MELAS may easily be misdiagnosed as AE, viral encephalitis, or cerebral infarction. Up to now, the pathophysiological mechanism of macroangiopathy in MELAS is not clear. Migratory vasodilation of cerebral arteries may be a specific MRI feature of MELAS. It needs to be emphasized that multimodal neuroimaging evaluation is very helpful in the diagnosis of mitochondrial diseases. We highly recommend dynamic MRA and ASL monitoring for MELAS patients. In MELAS, dilation of cerebral artery together with increased CBF not only suggests the clinical onset, but also implies an upcoming stroke-like attack.

## Data Availability

The original contributions presented in the study are included in the article/supplementary material. Further inquiries can be directed to the corresponding authors.
